# Why Current Publication Practices May Distort Science

**DOI:** 10.1371/journal.pmed.0050201

**Published:** 2008-10-07

**Authors:** Neal S Young, John P. A Ioannidis, Omar Al-Ubaydli

## Abstract

John Ioannidis and colleagues argue that the current system of publication in biomedical research provides a distorted view of the reality of scientific data.

This essay makes the underlying assumption that scientific information is an economic commodity, and that scientific journals are a medium for its dissemination and exchange. While this exchange system differs from a conventional market in many senses, including the nature of payments, it shares the goal of transferring the commodity (knowledge) from its producers (scientists) to its consumers (other scientists, administrators, physicians, patients, and funding agencies). The function of this system has major consequences. Idealists may be offended that research be compared to widgets, but realists will acknowledge that journals generate revenue; publications are critical in drug development and marketing and to attract venture capital; and publishing defines successful scientific careers. Economic modelling of science may yield important insights (Table 1).

## The Winner's Curse

In auction theory, under certain conditions, the bidder who wins tends to have overpaid. Consider oil firms bidding for drilling rights; companies estimate the size of the reserves, and estimates differ across firms. The average of all the firms' estimates would usually approximate the true reserve size. Since the firm with the highest estimate bids the most, the auction winner systematically overestimates, sometimes so substantially as to lose money in net terms [1]. When bidders are cognizant of the statistical processes of estimates and bids, they correct for the winner's curse by shading their bids down. This is why experienced bidders sometimes avoid the curse, as opposed to inexperienced ones [1–4]. Yet in numerous studies, bidder behaviour appears consistent with the winner's curse [5–8]. Indeed, the winner's curse was first proposed by oil operations researchers after they had recognised aberrant results in their own market.

**Summary**

The current system of publication in biomedical research provides a distorted view of the reality of scientific data that are generated in the laboratory and clinic. This system can be studied by applying principles from the field of economics. The “winner's curse,” a more general statement of publication bias, suggests that the small proportion of results chosen for publication are unrepresentative of scientists' repeated samplings of the real world. The self-correcting mechanism in science is retarded by the extreme imbalance between the abundance of supply (the output of basic science laboratories and clinical investigations) and the increasingly limited venues for publication (journals with sufficiently high impact). This system would be expected intrinsically to lead to the misallocation of resources. The scarcity of available outlets is artificial, based on the costs of printing in an electronic age and a belief that selectivity is equivalent to quality. Science is subject to great uncertainty: we cannot be confident now which efforts will ultimately yield worthwhile achievements. However, the current system abdicates to a small number of intermediates an authoritative prescience to anticipate a highly unpredictable future. In considering society's expectations and our own goals as scientists, we believe that there is a moral imperative to reconsider how scientific data are judged and disseminated.

An analogy can be applied to scientific publications. As with individual bidders in an auction, the average result from multiple studies yields a reasonable estimate of a “true” relationship. However, the more extreme, spectacular results (the largest treatment effects, the strongest associations, or the most unusually novel and exciting biological stories) may be preferentially published. Journals serve as intermediaries and may suffer minimal immediate consequences for errors of over- or mis-estimation, but it is the consumers of these laboratory and clinical results (other expert scientists; trainees choosing fields of endeavour; physicians and their patients; funding agencies; the media) who are “cursed” if these results are severely exaggerated—overvalued and unrepresentative of the true outcomes of many similar experiments. For example, initial clinical studies are often unrepresentative and misleading. An empirical evaluation of the 49 most-cited papers on the effectiveness of medical interventions, published in highly visible journals in 1990–2004, showed that a quarter of the randomised trials and five of six non-randomised studies had already been contradicted or found to have been exaggerated by 2005 [9]. The delay between the reporting of an initial positive study and subsequent publication of concurrently performed but negative results is measured in years [10,11]. An important role of systematic reviews may be to correct the inflated effects present in the initial studies published in famous journals [12], but this process may be similarly prolonged and even systematic reviews may perpetuate inflated results [13,14].

**Table 1:**
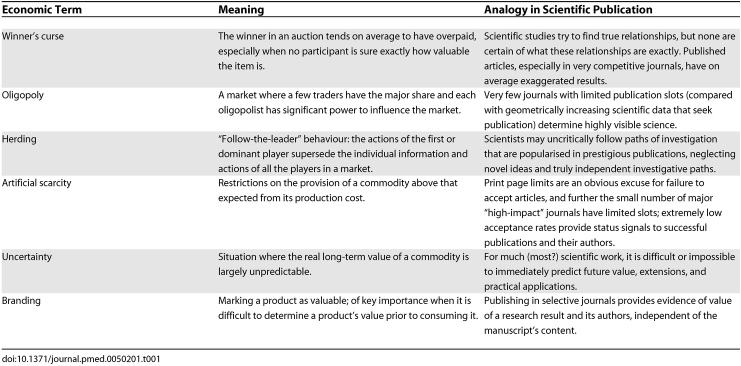
Economic Terms and Analogies in Scientific Publication

More alarming is the general paucity in the literature of negative data. In some fields, almost all published studies show formally significant results so that statistical significance no longer appears discriminating [15,16]. Discovering selective reporting is not easy, but the implications are dire, as in the “hidden” results for antidepressant trials [17]: in a recent paper, it was shown that while almost all trials with “positive” results on antidepressants had been published, trials with “negative” results submitted to the US Food and Drug Administration, with few exceptions, remained either unpublished or were published with the results presented so that they would appear “positive” [17]. Negative or contradictory data may be discussed at conferences or among colleagues, but surface more publicly only when dominant paradigms are replaced. Sometimes, negative data do appear in refutation of prominent claims. In the “Proteus phenomenon”, an extreme result reported in the first published study is followed by an extreme opposite result; this sequence may cast doubt on the significance, meaning, or validity of any of the results [18]. Several factors may predict irreproducibility (small effects, small studies, “hot” fields, strong interests, large databases, flexible statistics) [19], but claiming that a specific study is wrong is a difficult, charged decision.

In the basic biological sciences, statistical considerations are secondary or nonexistent, results entirely unpredicted by hypotheses are celebrated, and there are few formal rules for reproducibility [20,21]. A signalling benefit from the market—good scientists being identified by their positive results—may be more powerful in the basic biological sciences than in clinical research, where the consequences of incorrect assessment of positive results are more dire. As with clinical research, prominent claims sometimes disappear over time [21]. If a posteriori considerations are met sceptically in clinical research, in basic biology they dominate. Negative data are not necessarily different than positive results as related to considerations of experimental design, execution, or importance. Much data are never formally refuted in print, but most promising preclinical work eventually fails to translate to clinical benefit [22]. Worse, in the course of ongoing experimentation, apparently negative studies are abandoned prematurely as wasteful.

## Oligopoly

Successful publication may be more difficult at present than in the past. The supply and demand of scientific production have changed. Across the health and life sciences, the number of published articles in Scopus-indexed journals rose from 590,807 in 1997 to 883,853 in 2007, a modest 50% increase. In the same decade, data acquisition has accelerated by many orders of magnitude: as an example, the current Cancer Genome Atlas project requires 10,000 times more sequencing effort than the Human Genome Project, but is expected to take a tenth of the time to complete [23]. In the current environment, the distinction between raw data and articles (telling for sure what more an article has compared with raw data) can sometimes become difficult. Only a small proportion of the explosively expanded output of biological laboratories appears in the modestly increased number of journal slots available for its publication, even if more data can be compacted in the average paper now than in the past.

Constriction on the demand side is further exaggerated by the disproportionate prominence of a very few journals. Moreover, these journals strive to attract specific papers, such as influential trials that generate publicity and profitable reprint sales. This “winner-take-all” reward structure [24] leaves very little space for “successful publication” for the vast majority of scientific work and further exaggerates the winner's curse. The acceptance rate decreases by 5.3% with doubling of circulation, and circulation rates differ by over 1,000-fold among 114 journals publishing clinical research [25]. For most published papers, “publication” often just signifies “final registration into oblivion”. Besides print circulation, in theory online journals should be readily visible, especially if open access. However, perhaps unjustifiably, most articles published in online journals remain rarely accessed. Only 73 of the many thousands of articles ever published by the 187 BMC-affiliated journals had over 10,000 accesses through their journal Web sites in the last year [26].

Impact factors are widely adopted as criteria for success, despite whatever qualms have been expressed [27–32]. They powerfully discriminate against submission to most journals, restricting acceptable outlets for publication. “Gaming” of impact factors is explicit. Editors make estimates of likely citations for submitted articles to gauge their interest in publication. The citation game [33,34] has created distinct hierarchical relationships among journals in different fields. In scientific fields with many citations, very few leading journals concentrate the top-cited work [35]: in each of the seven large fields to which the life sciences are divided by ISI Essential Indicators (each including several hundreds of journals), six journals account for 68%–94% of the 100 most-cited articles in the last decade (Clinical Medicine 83%, Immunology 94%, Biochemistry and Biology 68%, Molecular Biology and Genetics 85%, Neurosciences 72%, Microbiology 76%, Pharmacology/Toxicology 72%). The scientific publishing industry is used for career advancement [36]: publication in specific journals provides scientists with a status signal. As with other luxury items intentionally kept in short supply, there is a motivation to restrict access [37,38].

Some unfavourable consequences may be predicted and some are visible. Resource allocation has long been recognised by economists as problematic in science, especially in basic research where the risks are the greatest. Rival teams undertake unduly dubious and overly similar projects; and too many are attracted to one particular contest to the neglect of other areas, reducing the diversity of areas under exploration [39]. Early decisions by a few influential individuals as to the importance of an area of investigation consolidate path dependency: the first decision predetermines the trajectory. A related effect is herding, where the actions of a few prominent individuals rather than the cumulative input of many independent agents drives people's valuations of a commodity [40,41]. Cascades arise when individuals regard others' earlier actions as more informative than their own private information. The actions upon which people herd may not necessarily be correct; and herding may long continue upon a completely wrong path [41]. Information cascades encourage conventional behaviour, suppress information aggregation, and promote “bubble and bust” cycles. Informational analysis of the literature on molecular interactions in Drosophila genetics has suggested the existence of such information cascades, with positive momentum, interdependence among published papers (most reporting positive data), and dominant themes leading to stagnating conformism [42].

## Artificial Scarcity

The authority of journals increasingly derives from their selectivity. The venue of publication provides a valuable status signal. A common excuse for rejection is selectivity based on a limitation ironically irrelevant in the modern age—printed page space. This is essentially an example of artificial scarcity. Artificial scarcity refers to any situation where, even though a commodity exists in abundance, restrictions of access, distribution, or availability make it seem rare, and thus overpriced. Low acceptance rates create an illusion of exclusivity based on merit and more frenzied competition among scientists “selling” manuscripts.

Manuscripts are assessed with a fundamentally negative bias: how they may best be *rejected* to promote the presumed selectivity of the journal. Journals closely track and advertise their low acceptance rates, equating these with rigorous review: “*Nature* has space to publish only 10% or so of the 170 papers submitted each week, hence its selection criteria are rigorous”—even though it admits that peer review has a secondary role: “the judgement about which papers will interest a broad readership is made by *Nature*'s editors, not its referees” [43]. *Science* also equates “high standards of peer review and editorial quality” with the fact that “of the more than 12,000 top-notch scientific manuscripts that the journal sees each year, less than 8% are accepted for publication” [44].

The publication system may operate differently in different fields. For example, for drug trials, journal operations may be dominated by the interests of larger markets: the high volume of transactions involved extends well beyond the small circle of scientific valuations and interests. In other fields where no additional markets are involved (the history of science is perhaps one extreme example), the situation of course may be different. The question to be examined is whether published data may be more representative (and more unbiased) depending on factors such as the ratio of journal outlets to amount of data generated, the relative valuation of specialty journals, career consequences of publication, and accessibility of primary data to the reader.

One solution to artificial scarcity—digital publication—is obvious and already employed. Digital platforms can facilitate the publication of greater numbers of appropriately peer-reviewed manuscripts with reasonable hypotheses and sound methods. Digitally formatted publication need not be limited to few journals, or only to open-access journals. Ideally, all journals could publish in digital form manuscripts that they have received and reviewed and that they consider unsuitable for print publication based on subjective assessments of priority. The current privileging of print over digital publication by some authors and review committees may be reversed, if online-only papers can be demonstrated or perceived to represent equal or better scientific reality than conventional printed manuscripts.

## Uncertainty

When scientific information itself is the commodity, there is uncertainty as to its value, both immediately and in the long term. Usually we do not know what information will be most useful (valuable) eventually. Economists have struggled with these peculiar attributes of scientific information as a commodity. Production of scientific information is largely paid for by public investment, but the product is offered free to commercial intermediaries, and is culled by them with minimal cost, for sale back to the producers and their underwriters! An explanation for such a strange arrangement is the need for branding—marking the product as valuable. Branding may be more important when a commodity cannot easily be assigned much intrinsic value and when we fear the exchange environment will be flooded with an overabundance of redundant, useless, and misleading product [39,45]. Branding serves a similar and complementary function to the status signal for scientists discussed above. While it is easy to blame journal editors, the industry, or the popular press, there is scant evidence that these actors bear the major culpability [46–49]. Probably authors themselves self-select their work for branding [10,11,50–52].

## Conclusions

We may consider several competing or complementary options about the future of scientific publication (Box 1). When economists are asked to analyse a resource-allocation system, a typical assumption is that when information is dispersed, over time, the individual actors will not make systematic errors in their inferences. However, not all economists accept this strong version of rationality. Systematic misperceptions in human behaviour occur with some frequency [53], and misperceptions can perpetuate ineffective systems.

**Box 1. Potential Competing or Complementary Options and Solutions for Scientific Publication**

1. Accept the current system as having evolved to be the optimal solution to complex and competing problems.
2. Promote rapid, digital publication of all articles that contain no flaws, irrespective of perceived “importance”.
3. Adopt preferred publication of negative over positive results; require very demanding reproducibility criteria before publishing positive results.
4. Select articles for publication in highly visible venues based on the quality of study methods, their rigorous implementation, and astute interpretation, irrespective of results.
5. Adopt formal post-publication downward adjustment of claims of papers published in prestigious journals.
6. Modify current practice to elevate and incorporate more expansive data to accompany print articles or to be accessible in attractive formats associated with high-quality journals: combine the “magazine” and “archive” roles of journals.
7. Promote critical reviews, digests, and summaries of the large amounts of biomedical data now generated.
8. Offer disincentives to herding and incentives for truly independent, novel, or heuristic scientific work.
9. Recognise explicitly and respond to the branding role of journal publication in career development and funding decisions.
10. Modulate publication practices based on empirical research, which might address correlates of long-term successful outcomes (such as reproducibility, applicability, opening new avenues) of published papers.

Some may accept the current publication system as the ideal culmination of an evolutionary process. However, this order is hardly divinely inspired; additionally, the larger environment has changed over time. Can digital publication alleviate wasteful efforts of repetitive submission, review, revision, and resubmission? Preferred publication of negative over positive results has been suggested, with print publication favoured for all negative data (as more likely to be true) and for only a minority of the positive results that have demonstrated consistency and reproducibility [54]. To exorcise the winner's curse, the quality of experiments rather than the seemingly dramatic results in a minority of them would be the focus of review, but is this feasible in the current reality?

There are limitations to our analysis. Compared with the importance of the problem, there is a relative paucity of empirical observations on the process of scientific publication. The winner's curse is fundamental to our thesis, but there is active debate among economists whether it inhibits real environments or is more of a theoretical phenomenon [1,8]. Do senior investigators make the same adjustments on a high-profile paper's value as do experienced traders on prices? Is herding an appropriate model for scientific publication? Can we correlate the site of publication with the long-term value of individual scientific work and of whole areas of investigation? These questions remain open to analysis and experiment.

Even though its goals may sometimes be usurped for other purposes, science is hard work with limited rewards and only occasional successes. Its interest and importance should speak for themselves, without hyperbole. Uncertainty is powerful and yet quite insufficiently acknowledged when we pretend prescience to guess at the ultimate value of today's endeavours. If “the striving for knowledge and the search for truth are still the strongest motives of scientific discovery”, and if “the advance of science depends upon the free competition of thought” [55], we must ask whether we have created a system for the exchange of scientific ideas that will serve this end.

## Supporting Information

Text S1 An extended version of this analytical essay (115 KB PDF).
