# Supplementary material for: Why Current Publication Practices May Distort Science
Source: PLoS Med. 2008 Oct 7;5(10):e201. doi: 10.1371/journal.pmed.0050201 (PMC2561077; doi:10.1371/journal.pmed.0050201)
Supplement: Supplementary file 1 [file pmed.0050201.sd001.pdf]

# Why Current Publication Practices May Distort Science. The Market for Exchange of Scientific Information: The Winner's Curse, Artificial Scarcity, and Uncertainty in Biomedical Publication

**Neal S. Young**[*1], **John P. A. Ioannidis**[2], **Omar Al-Ubaydli**[3]

**Summary.** The current system of publication in biomedical research provides a distorted view of the reality of scientific data that are generated in the laboratory and clinic. This system can be studied by applying principles from the field of economics. The "winners curse", a more general statement of publication bias, suggests that the small proportion of results chosen for publication are unrepresentative of scientists repeated samplings of the real world. The self-correcting mechanism in science is retarded by the extreme imbalance between the abundance of supply (the output of basic science laboratories and clinical investigations) and the increasingly limited venues for publication (journals with sufficiently high impact). This system would be expected intrinsically to lead to the misallocation of resources. The scarcity of available outlets is artificial, based on the costs of printing in an electronic age and a belief that selectivity is equivalent to quality. Science is subject to great uncertainty: we cannot be confident now which efforts will ultimately yield worthwhile achievements. However, the current system of publication abdicates to a small number of intermediates an authoritative prescience to anticipate a highly unpredictable future. In considering societys expectations and our own goals as scientists, we believe that there is a moral imperative to reconsider how scientific data are judged and disseminated.

*Science can be viewed from various standpoints, not only from that of epistemology: for example, we can look at it as a biological and or as a sociological phenomenon. As such, it might be described as a tool, or an instrument, comparable perhaps to some of our industrial machinery. Science may be regarded as a means of production...*

*Karl Popper, The Logic of Scientific Discovery, 1932 (1)*

*The central economic fact about the processes of invention and research is that they are devoted to the production of information.*

*Kenneth Arrow, Economic welfare and the allocation of resources for invention, 1962 (2)*

[1]Hematology Branch, National Heart, Lung, and Blood Institute, National Institutes of Health, Bethesda, Maryland, United States of America

[*]to whom correspondience should be addressed at CRC-3E-5140, NIH, Bethesda MD 20892-1202; 301 496 5093; youngns@mail.nih.gov

[2]Department of Hygiene and Epidemiology, University of Ioannina School of Medicine, Ioannina, and the Biomedical Research Institute, Foundation for Research and Technology Hellas, Ioannina, Greece; Department of Medicine, Tufts University School of Medicine Boston, Massachusetts, United States of America

[3]Department of Economics and the Mercatus Center, George Mason University, Fairfax, Virginia, United States of America

An underlying assumption of this essay is that science creates a commodity susceptible to economic thinking. Scientific information is a product that is traded in the market of scientific journals. How well this market functions–or misbehaves–can have major consequences. Qualitative and quantitative aspects of the selection process for scientific data to be disseminated can be viewed using principles from the field of economics. Our system of publication has clear historical origins, and likely from its beginnings several centuries ago it has served multiple purposes. Certainly, an explicit aim at the outset and presumably still the main goal is the advancement of science, or more concretely the materialization, assessment, and dissemination of scientific data and ideas; secondary aims have also evolved, which include the career needs of the scientific community or the business plans of journals.

Idealists at the bench or bedside may be offended that their research be compared to widgets. Realists will acknowledge that there are multiple valuations of well published data. Journals generate revenue for commercial scientific publishers and nonprofit scientific societies. Publication of data is critical in drug development and marketing for the pharmaceutical industry and to attract investment and venture capital to biotechnology companies. The publication record of individuals and of whole scientific fields powerfully affects the allocation of public grants and private donations and the direction of funding supports to promising avenues of research. For investigators themselves, the quantity and quality of an individuals publications and citations is basic to the incentive system that motivates and rewards a scientific career: getting results into print is a symbolic equivalent for making a significant discovery (3). Publication is perhaps the major factor in hiring and tenure decisions, in the acquisition of promotions and honors, and in the very definition of a successful career.

Economists also may offer objections to the proposed analysis, arguing that the "marketplace for ideas" is only metaphoric and that key defining features of a market are not present, or at least not easily recognized, in the process of making public the results of scientific research. How well does this system work? As a question in classical welfare economics, to what extent does competition lead to optimal allocation of resources? Modern economists have productively addressed "science as a social institution" (3, 4) in narrative and quantitative critiques of the allocation of resources for scientific research (5, 2, 6) and in descriptions of the economic characteristics of scientific knowledge (7, 8); of sciences reward structure and incentives (3, 9, 10) and predictors of productivity (3, 11, 12); the capacity of societies to translate scientific knowledge into economic benefits (13), and the risks of research as they apply to science as a private enterprise versus a public good (5, 14, 15), to name a few examples. For this essay, to aid the reader we have provided definitions of some potentially unfamiliar terms from economics and a brief summary of their applicability to current publishing material in Table 1.

Science is our most widely accepted, least subjective, and best organized method of understanding the world. Understanding the structure and function of genes, molecules, cells, and organisms, the complexities of interactions among these entities, and ultimately beneficial medical advances all are speeded (or slowed) by the efficiency of the system of publication–publishing in the literal sense of making public and known. We

argue here that our critique has implications for the accuracy and therefore the reliability of the current system of publication because of the highly selective presentation of the world of experimental laboratory and clinical data. We will further suggest that, even in the absence of normative standards and some of the difficulties of treating the complex scientific enterprise as a simple marketplace, concrete methods to investigate and to ameliorate issues of market inefficiency may be considered.

*Our understanding of inventive activity...is excessively rooted in success stories.*

*Nathan Rosenberg, Science, invention, and economic growth, 1974 (16)*

## The "winner's curse" and publication bias

The winner's curse is a familiar principle in auction theory: the bidder who wins an auction has a tendency to have overpaid (because the real market value of an item is best estimated by the mean of all bids). An historical exampleindeed the origin of the winners curse principle in economics– is oil firms bidding for drilling rights; each company will have employed its own geologists to estimate the size of the reserves, and these estimates will differ across firms. These auctions are said to have common uncertain values (bidders similarly value the item but are uncertain of its true worth). The winners curse follows from the tendency for the winner of the auction to systematically overestimate, sometimes so substantially as to lose considerable monetary sums in net terms (17). The average of all the firms estimates would usually represent a good estimate of the reserves size. Since firms with higher estimates bid higher amounts, the firm with the highest estimate (rather than the average estimate) will win the auction. When the number of bidders increases, the chances of a winner being cursed are typically higher and the curse (overestimate) greater. If bidders are cognizant of the statistical processes that underlie each bidders estimates and bids, they can correct for a winners curse by shading their bids down. In other words, the winners curse represents systematic errors by the bidders, to which rational bidders should be less susceptible (18). Inexperienced auction participants appear particularly prone, while experienced bidders participating in familiar, professional auctions do not suffer from the winners curse (19, 20). Difficult as it may be to definitively demonstrate systematic errors by bidders, in numerous studies bidder behavior is consistent with features of the winners curse (21-24). Indeed, the winners curse was first proposed by oil operations researchers after they had recognized aberrant results in their own market.

The winners curse principle can be applied to the publication of scientific data in general and in biomedicine in particular. Although manuscript authors often feel like supplicants, they are offering to the journals material of enormous value, to the authors, the journals, and the wider public. The journals function as bidders should be cursed if they acquire an over-valued commodity, an unrepresentative result or a misleading paper. That they are not is a reflection of many aspects of the system (to be discussed below, but including the oligopoly of the few high impact factor journals, the journals distinct commercial and social goals, and the role of the journals as intermediaries for the transmission of information to the public). So those cursed are the ultimate consumers of published information, which include some authors (only a few of whom are expert in a particular field) but mainly other scientists,

physicians, patients, funding agencies, newspaper science writers, hiring, tenure and promotion committees. As consumers, to visualize the winners curse in the context of scientific research, imagine many laboratories or clinics each sampling reality in order to answer a specific question. Our view of scientific reality is potentially distorted if we only learn the results of a nonrandom few of these investigations, those describing the most spectacular results. Such data are unlikely to be representative of the more mundane findings from the "average" laboratory studying the same problem. As a result, the real value of the most striking findings is potentially overestimated (and later overstated).

The principle of the winners curse is more general and extensive than the problem of publication bias. The winner's curse expresses more than a preference for manuscripts with low p-values. Nevertheless, based solely on favoritism for a stronger statistical result, it has been recognized for decades that the initial pilot clinical trial or the first published genetic linkage analysis is highly likely to be unrepresentative and very probably misleading. Highly visible clinical research findings often are contradicted or found to be exaggerated by subsequent research on the same questions (25). An empirical evaluation of the 49 most-cited papers on the effectiveness of medical interventions, published in highly visible journals in 1990–2004, showed that a quarter of the randomized trials and five of six nonrandomized studies had already been contradicted or found to have been exaggerated by 2005 (25). If comparable studies simply appeared in rapid sequence based on their statistics, a short time-lag bias might be acceptable. Unfortunately, systematic study of the biomedical literature is not encouraging in this respect. The delay between the reporting of an initial positive study and subsequent publication of concurrently performed but negative results is measured in years (26, 27). An important role of systematic reviews may be to correct the inflated effects presented in the initial studies published in famous journals (28), but the correction may take a long time and even systematic reviews may reflect inflated effects (29,30).

More alarming is the general paucity in the literature of negative data. In some fields, almost all published studies show formally significant results, so that statistical significance no longer appears discriminating (31,32). Discovering that a series of overwhelmingly positive published trials is only a selection from a larger data set is not easy, but the implications, especially in clinical medicine, are dire, as in the recent example of "hidden" results of clinical trials for antidepressant efficacy (33): in a recent paper, it was shown that while almost all trials with "positive" results on antidepressants had been published, trials with "negative" results submitted to the US Food and Drug Administration, with few exceptions, remained either unpublished or were published with the results presented so that they would appear "positive". Negative or contradictory data may be discussed at conferences or among colleagues but surface more publicly only when one dominant paradigm is replaced by another. Sometimes, negative data do appear in refutation of a prominent positive study. In the "Proteus phenomenon", an extreme result reported in the first published study is followed by an extreme opposite result; this sequence may cast doubt on the significance, meaning, or validity of any of the results (34). Several factors may predict irreproducibility, including small effects, small studies, "hot" fields with strong interest in specific results, large databases, and flexible statistics (35), but claiming that a specific study is wrong is a difficult, charged decision. Diverse and se-

rious attempts have been made to address these problems in clinical research: emphasis on a priori hypothesis testing, mandatory registration of clinical protocols, the scrutiny of protocols for primary endpoints to avoid "significance-seeking", and statistical solutions to missing data are examples (36–41).

In the basic biological sciences, in which statistical considerations often are secondary or nonexistent, results entirely unpredicted by hypotheses are celebrated, and there are few formal rules for reproducibility despite the essentially inductive nature of most biological laboratory investigations (42, 43). A signaling benefit from the marketgood scientists being identified by their positive resultsmay be more powerful in the basic biological sciences than in clinical research, where the consequences of incorrect assessment of positive results are more dire. As with clinical research, large effects and prominent claims sometimes gradually disappear over time as data accumulate (43). If *a posteriori* considerations are met relatively skeptically in clinical research, in basic biology they dominate, even though negative data are not necessarily different from positive results as related to experimental design, execution, and interpretation, or their ultimate importance. Much data are never formally refuted in print, but the overwhelming majority of promising preclinical work eventually fails to translate to clinical benefit (44). Most published research claims may not be true (35).

As will be further argued below, an inherent bias to publish positive data has been exacerbated in the current market for biologic and medical information because of an increasing imbalance between supply (the cumulative output of scientific laboratories) and demand (the space available in prominent scientific journals). Following from the winners curse, journals in general and especially those most selective in their acceptance rates will be far more likely to publish certain types of data than others, results that are strikingly positive, or unexpected, or which carry the promise of commercial or biotechnology exploitation, or those likely to be reported to the general public in the mass media. Regardless of equivalence in the quality of initial hypotheses, experimental design, execution, and interpretation, results describing modest outcomes or negative and unspectacular data are difficult to publish in selective journals. As a result, this set of data–likely the majority of the output of scientific laboratories,–will either never be printed or appear in lightly regarded outlets and, worse, in the course of ongoing experimentation, apparently negative studies will be abandoned prematurely as wasteful.

*...even the trade journal must, unless the product of collective action, be somebodys business.*

*Joseph Schumpeter, The Instability of Capitalism, 1928 (45)*

## Information as a commodity: matching agents and oligopoly

If the winner's curse only applied to the speed of publication it might be largely irrelevant; most scientists express faith in the self-correcting nature of science. However, successful publication, in the sense both of appearance in a prestigious journal as well as broadcasting and acceptance of research data, may actually be more difficult at present than in the past. If "the central economic fact about the processes of invention and research is that they are devoted to the production of information" (2), what are the consequences in the market of this altered environment? One first re-

quirement is to acknowledge that neither the supply nor the demand sides of the simple equation of scientific production and publication is static. While the number of scientific journals has certainly multiplied, this increase has not paced the explosion in scientific productivity. Across the health and life sciences, the number of published articles in Scopus-indexed journals rose from 590,807 in 1997 to 883,853 in 2007, a modest 50% increase. In the same decade, data acquisition has accelerated far more: as an example, the current Cancer Genome Atlas project requires 10,000 times more sequencing effort than the Human Genome Project, but is expected to take a tenth of the time to complete (46). Historically, a scientist in training might have required a year to purify and characterize a protein in the laboratory; clinical investigation focused on small numbers of modest sized protocols. Contrast this primitive recent past with the huge expansion in the biomedical research community and dramatic improvements in productivity: recent doubling of the NIH budget and expenditure of enormous sums by the pharmaceutical and biotechnology industries combined with new methodologies (such as genome based techniques), developments of commercial kits (especially in molecular biology), and availability of contracting facilities to efficiently outsource complex methods. Petabytes of biologic and medical information currently are generated in thousands of laboratories worldwide, orders of magnitude more information in comparison to a few decades ago. However, only a small proportion of this expanded output appears in a modestly greater number of journals available for its publication. Even if more data can be now compacted in the average paper than in the past, much information is either never seen or fails to satisfy a small number of peer reviewers and editors, and ultimately is never distributed to scientists, physicians, patients and the public.

Constriction on the demand side is further exaggerated by the disproportionate prominence of a very few journals. Indeed, the very success of these preeminent journals may be due to the creation of a "buyers' market" from a surplus of excellent raw material, the output of biomedical laboratories, universities, and research organizations. Moreover, these journals strive to attract specific papers, such as influential trials that generate publicity and profitable reprint sales. This leaves very little space for "successful publication" for the vast majority of scientific work and further exaggerates the winner's curse. The acceptance rate decreases by 5.3% with doubling of circulation, and circulation rates differ by over 1,000-fold among 114 journals publishing clinical research (47). For most published papers, "publication" often just signifies "final registration into oblivion". As an alternative to print circulation, online journals should be more readily visible, especially if open access. However, perhaps unjustifiably and maybe only temporarily, most articles published in online journals remain rarely accessed. Only 73 of the many thousands of articles ever published by the 187 BMC-affiliated journals had over 10,000 accesses through their journal websites in the last year (48).

This market imbalance exaggerates the "winner-take-all" reward structure of science, a strategy that translates small differences in human capital into much larger differences in the distribution of economic award (49). How well does this system work towards the goal of efficient advancement of biomedical knowledge? Producing scientists are aware that the outlet for their work is controlled by a small number of editors whose qualifications, biases, and interests - to the extent that can be inferred- may differ substantially from those of active investigators in the laboratory and clinic. Edi-

tors rely on reviewers, but their selection by the journal, the interpretation of their comments by an editorial board, and the (largely anonymous) reviewers own backgrounds, biases and competing interests introduce substantial subjectivity and latitude in formulating the final decision to publish. Journals as commercial enterprises pursue their own goals, which need not match perfectly those of the scientific community, the funding agencies, and the public that both sponsors and ultimately relies upon the scientific enterprise.

Impact factors, which measure the frequency of citations from a journals' publications, served first to discriminate among journals, but they are now widely adopted as criteria for success by both the publishing houses and submitting authors, despite qualms concerning their calculation and meaning (50-55). They serve to powerfully discriminate against submission to the vast majority of scientific journals, restricting acceptable outlets for publication. Given the importance of impact factors, their "gaming" is not only inevitable but often explicit. Editors make estimates of likely citations for submitted articles to gauge their interest in publication, and prospective authors strive to increase the visibility of their work by publication in a very few journals that can greatly influence their scientific credibility, career tracks, and their institutions prestige. Indeed, impact factors and the related citation game have created dubious criteria for acceptance of manuscripts (56, 57) and distinct hierarchical relationships among journals in different fields of biomedicine. In scientific fields with many citations, very few leading journals concentrate the large majority of the top-cited work (58): in each of the 7 large fields to which the life sciences are divided by ISI Essential Indicators (each including several hundreds of journals), 6 journals account for 68-94% of the 100 most-cited articles in the last decade (Clinical Medicine 83%, Immunology 94%, Biochemistry and Biology 68%, Molecular Biology and Genetics 85%, Neurosciences 72%, Microbiology 76%, Pharmacology/Toxicology 72%). The entire scientific publishing industry has been unapologetically utilized for the purpose of career advancement (59): publication in specific journals provides to scientists a status signal, and similar to other luxury items intentionally restricted to short supply, the consequences may include both the payment of a high premium for the same utility and a strong motivation to restrict access (60, 61).

Some not entirely favorable expectations of this system may be predicted and indeed some are visible. Resource allocation has long been recognized by economists as problematic in science, especially in basic research where the risks may be perceived as greatest. Competition among scientists for very limited rewards can cause wastage of resources, as rival teams undertake unduly dubious or overly similar projects; and too many are attracted to one particular contest to the neglect of other fields (8). This process reduces the diversity of scientific areas under exploration that become susceptible to actual selection on a more open market. Early decisions (made by journal editors) as to the importance of an area of investigation would be predicted to result in the creation and consolidation of acquired advantages– in economic terms, path dependency, in which the first decision predetermines the trajectory.

A related effect likely to be promoted by the manuscript selection process of elite journals is herding, in which the actions of a few prominent individuals rather than the cumulative input of many independent agents drive a market (62, 63). Herding is favored by circumstances in which sequential discrete decisions are made with the knowledge of the actions, but not the motives, of other actors;

"follow the leader" behavior diminishes the value of a market as based on summing of many private signals to maximize total information. In other words, the actions of imitators is uninformative to those who decide later, and the consequence can be information blockage and information cascade. Ordinarily, market efficiency depends on many individuals using their private information to make informed decisions. However, cascades arise when individuals regard others earlier actions as more informative than their own private information, and each individual ignores his own private information and simply imitates the actions of those acting before him. Unfortunately, "the general conclusion that there can be long periods in which individuals herd upon poor decisions is robust" (63). Information cascades lead to inefficient marketplaces: they affect especially the decision to participate, they encourage conventional behavior, and they suppress or slow information aggregation. In commercial markets, these effects promote disastrous "bubble and bust" cycles, despite clear goals (profits), transparency (pricing), and no formally dominant gatekeepers (for entry into the market)-circumstances in some contrast to the more opaque (and thus even more vulnerable) system of scientific publication.

Informational analysis of a restricted literature on molecular interactions in Drosophila genetics has suggested the existence of such information cascades, with evidence of positive momentum, a high degree of interdependence among published papers with most reporting positive data, and dominant themes rapidly leading to stagnating conformism (64). Historical and theoretical evidence suggests that the "logic of private science... transforms itself into a powerful machine for constructing irreversibility and limiting the variety of technological options or the range of possible choices" (15). Empiric testing and formal economic modeling would provide a more accurate view of the effects on market efficiency of the current publication system.

### Artificial scarcity and the problem of "surplus"

The influence and authority of journals is perceived to increasingly derive, not from any demonstrable evidence of superior reviewers and editors, but from their selectivity. It is the venue of publication, rather than the substance of the work, that provides a valuable status signal, as discussed above. The basis for selection and rejection at major journals often appears highly subjective: manuscripts are returned after brief editorial review as not of interest to the journal, or rejected after favorable peer review as lacking sufficiently high impact. Absent a normative standard, a common excuse is selectivity based on a limitation ironically irrelevant in the modern age–printed page space. In economical terms, this is essentially an example of artificial scarcity. Artificial scarcity refers to any situation in which, even though a commodity exists in abundance, restrictions of access, distribution, or availability make it seem rare, and thus liable to be overpriced. Publishers benefit from this false limitation, trivially in savings of page charges but powerfully in the generation of low acceptance rates. Low acceptance rates create an illusion of exclusivity based on merit and more frenzied competition among scientist "sellers" of manuscripts. Page limits drive an often preposterous process in which manuscripts are assessed with a fundamentally negative bias: how they may best be rejected in order to promote the presumed rigor of the review process and the selectivity of the journal. Journals closely track and advertise their low acceptance rates, equating them with rigorous review: "*Nature* has space to publish only 10% or so of the 170 papers submitted each week, hence its se-

lection criteria are rigorous" – even though it admits that peer-review has a secondary role: "the judgement about which papers will interest a broad readership is made by *Nature*'s editors, not its referees."(65) Science also equates "high standards of peer review and editorial quality" with the fact that "of the more than 12,000 top-notch scientific manuscripts that the journal sees each year, less than 8% are accepted for publication."(66).

The publication system may operate differently in different fields. For example, for drug trials, journal operation may be dominated by the interests of larger markets: the high volume of transactions involved extends well beyond the small circle of scientific valuations and interests. In other fields where no additional markets are involved (the history of science perhaps one extreme example), the situation of course may be different. Worth examining is whether published data may be more representative (and more unbiased) depending on factors such as the ratio of journal outlets to amount of data generated, the relative valuation of specialty journals, career consequences of publication, and the accessibility of primary data to the reader.

Of course, one solution to artificial scarcitydigital publication–is obvious and already employed. Most scientists and physicians must now consume scientific data mainly in digital format, as abstracts and full articles more readily accessed via the internet than available in the dusty stacks of medical libraries. The Public Library of Science was founded on a digital platform, with its main goal was to make accessible to a wide audience publicly funded medical and basic research. Arguments have centered on the viability of such open publishing enterprises and the effects of competition on the traditional publishing industry. In theory, digital platforms can facilitate the publication of greater numbers of appropriately peer-reviewed and carefully edited manuscripts describing work based on reasonable hypotheses and utilizing sound methods, with less regard for results fulfilling subjective and transient criteria based on hyperbole and speculation. Digitally formatted publication need not be limited to select journals or only to open access journals. Ideally, all journals could publish in digital format manuscripts that they have received and reviewed and that they consider unsuitable for print publication based on subjective assessments of priority.

A fear associated with the unsupervised character of the internet is indiscriminate publication of unfiltered results-"data dumping"–among consumers already suffering information overload. In some fields, papers do become available as pre-prints without any peer-review, especially in the physical sciences, in which all the primary data can be more readily presented by the authors and assessed by readers ArXiv already contains more than 400,000 preprints). In other fields (economics among them), the practice is to publish working papers, in which readers function as peer reviewers, and in which the goal may not be the finished publication itself. Similar, small scale efforts at unsupervised publication and the introduction of open threads recently have been initiated in the biomedical sciences. However, neither wider nor more immediate access to unscrutinized raw data nor abandonment of peer review is implied by the current analysis. The current privileging of print over digital publication by some authors and review committees may be reversed, if online-only papers can be demonstrated or perceived to represent equal or better scientific reality than conventional printed manuscripts.

*And surely you will easily believe that we have so many things truly natural which induce*

*admiration, could in a world of particulars deceive the senses, if we would disguise those things and labour to make them to seem more miraculous. But we do hate all impostures and lies; insomuch as we have severely forbidden it to all our fellows, under pain of ignominy and fines, that they do not show any natural work or thing, adorned or swelling, but only pure as it is, without affectation of strangeness.*

*Francis Bacon, The Great Instauration and New Atlantis, 1521 (67)*

## Defining a market when uncertainty dominates

When scientific information itself is the commodity, there is great uncertainty as to the value, both immediately and in the long-term, of this product. With uncommon exceptions, we really do not know what scientific information will be most useful and therefore valuable in the future. Uncertainty is easily exemplified in the biomedical sciences by the small number of actual translations into clinical applications (44): very few explicitly promised and much touted breakthroughs actually materialize in practice. Economists have struggled with these peculiar attributes of information as a commodity. One major issue is the risk in investment in gathering scientific information and the difficulty of providing adequate incentives: "...basic research, the output of which is only used as informational input for other inventive activities, is especially unlikely to be rewarded" (2). Debates have occurred over the value and applicability of market systems, the unfettered outcomes of the combined activities of individuals pursuing their own ends, and the role of regulation and the imposition of controls based on *a priori* principles of behavior. As applied to science, invention, and technology, arguments have been made for both liberal and interventional allocation of resources, the assignment of incentives and rewards, and the means to convert information into economic benefits, to convert knowledge into social goods.

We can only guess at Adam Smiths critique of a system in which a commodity, the production of which is largely paid for by public investment, is offered free of cost to commercial intermediaries who have no particular obligations to either the investors or producers. The product is culled by them, again at minimal cost, for sale back to the producers and their underwriters! Surely one–perhaps the major–explanation for such a strange arrangement is the need for branding, for marking the product as valuable, when its value is unclear. (Indeed, a commodity with immediately identifiable value may not require publication other than through the patent or drug approval process.) Branding may be more important when a commodity cannot easily be assigned much intrinsic value (immediate or long-term) and when there is a fear that the market will be flooded with an overabundance of redundant, useless, and misleading products (3, 8). Branding serves a similar and complementary function to the status signal for scientists discussed above; both serve tasks separate from the unbiased dissemination of scientific information.

The process of publication itself may be the major real or potential cause of bias in reporting (68-70). While no doubt there is a considerable appeal to lay collective blame for current dissatisfactions to anonymous journal editors (the sources of a stream of endless rejection letters), to the pharmaceutical and biotechnology industry (an eternally convenient target) or to the foibles of the popular press, there is scant evidence that any of these actors bear the major culpability (71-75). Conversely it may be the

community of authors themselves who self-select their work for presentation (7,1, 26, 27,75, 76).

*Science is a public good, which must be preserved at all costs because it is a source of variety. It causes new states of the world to proliferate. And this diversity depends on the diversity of interests and projects that ... reconfigure nature and society. Withoutthis source of diversity, the market–with its natural propensity to transform science into a commodity-would be ever more doomed to convergence and irreversibility.*

*Michel Callon, Is science a public good?, 1993 (15)*

## Conclusions

We may consider several competing or complementary options as to the future of scientific publication (Table 2). When asked to analyze a resource allocation system, the standard approach in economics is to identify the systems controllable parameters and to then deduce their optimal values given the goals of the various actors. These optimal values are then compared to the current state of the systems parameters, and recommendations for changes are presented. A key assumption in the analysis is that when information is dispersed, over time, the individual actors do not make systematic errors in their inferences. Avoiding systematic errors differs from not making errors -rather, individuals optimally process the information at their disposal to make unbiased predictions about the unknown.

Economists do have faith that resource-allocation systems will self-correct, especially when the cost to the actors of coordinating their actions is relatively low. This view is the natural extension of the principle that people typically act in their own best interest. Key to the belief that systems tend to self-correct is an absence of systematic errors, as discussed above. Active improvement of a system is far more likely to be effective when its actors fully understand its mechanics and the implications of changing its parameters. However, not all economists accept this strong version of rationality. Moreover there is a substantial psychology literature documenting systematic misperceptions in human behavior (77) - misperceptions that can may often perpetuate ineffective systems. Observational and experimental studies in economics have demonstrated the role of experience in the attenuation of systematic errors and misperceptions by experience (78) but also the often troubling persistence of such errors in markets.

In this essay, we have suggested explanations for why the current system of publication in biology and medicine may misrepresent the real world of experimentation in the laboratory and clinic. That misrepresentation is a real problem reflected in decades old concern with publication bias in clinical research and has recent empirical support (35, 43, 59, 44, 79 , 25, 34). If the provision of accurate information is the goal, the marketplace for scientific ideas is inefficient and unnecessarily prone to error. We conclude with several possible implications.

First, we must acknowledge limits to our analysis. Compared with the importance of the problem, there is a relative paucity of empirical observations related to the process of publication in the sciences. Practicing scientists are notoriously uninterested in the history and philosophy of science, and potentially resistant to an economics approach to their craft and careers. Nevertheless, in suggesting the role of certain economic principles, we can only approximate their impor-

tance in biomedicine. The winners curse is fundamental to our thesis, but there is active debate among economists as to whether in practice it inhibits real markets or is a phenomenon best observed in the classroom or behavioral laboratory (24, 17). Do senior investigators make the same adjustments on a high profile papers value as do experienced traders for the curses effect on prices, and is their calculation accurate? Information cascades appear to operate in some of the biology literature; is herding an appropriate model for scientific publication and its consequences? And can we assess correlation between the site of publication and the long-term value of individual scientific work and indeed of whole areas of investigation? These questions are open to analysis and experiment.

Second, there are immediate solutions to some aspects of the winners curse and the related phenomena we describe, many of which can be the subject of “experimentation” in the publishing industry. Scientists, editors, and publishers willingly accept the current system to market data as ideal, the consequence of an evolutionary, organic process. However, this order is hardly divinely inspired and, as we indicated frequently above, may operate as a highly imperfect, even flawed market; additionally, the larger environment in which it functions has changed dramatically over time. Rather, understanding the underlying forces allows a critical analysis of the consequences of the current system and potentially desirable changes. Open access journals, although motivated by other reasons, are largely digital; their fiscal viability remains uncertain. Some journals are attempting publication of biologic data without review, as in physical sciences and mathematics, with threads of commentary and criticism invited, as in the social sciences. Will these new efforts attract an audience of authors and readers? Might digital publication alleviate the often wasteful effort of repetitive submission, review, revision, and resubmission, the method and goal of which is a high visibility venue? Preferred publication of negative over positive results has been suggested, with print publication favored for all negative data (as more likely to be true) and for only a minority of the positive results that have demonstrated consistency and reproducibility (80). To exorcise the winners curse, the quality of experiments rather than the seemingly dramatic results in a minority of them would be the goal of review, but only a rare journal advertises its added value to be the quality and rigor of their editorial and review process rather than their ability to attract large numbers of submissions and reject most of them. Digital capabilities should allow a journal to publish all sound manuscripts, regardless of their results. Other mechanisms to allow the private consumer to extract the personally interesting and useful from increasing and daunting amounts of information include summaries, reviews, and digests. The basic scientists may learn from the structured meta-analytic review of the clinician, and the clinician may rediscover the value of the critical analytic narrative review.

Third, as basic scientists and clinical investigators, we extol the value of knowledge even when its practical consequences are uncertain. Recognition of the economic forces operating in the dissemination of scientific data, and the resulting distortion of the reality of experimental results as published, is important. Do the structural features of the current system of publication favor the appropriate goals? Are these goals explicit for those who produce, consume, and fund science? Is a more efficient, “liberal” market conceivable, desirable, and practical? Can we direct attention to the substance of published work rather than the provenance of its appearance? Can we direct attention to the

underlying reasons for faddish trends, false hopes and alarms? Can we influence the already fraught process of allocation of public and private resources? If we accept science as a public good, is there a moral imperative, similar to that of equipoise in clinical trials, to the fuller, less biased presentation of the work of publicly funded laboratories? An economic analysis should not be interpreted as an attack on the scientific enterprise itself: however imperfect and often difficult to define, science is the only method we have to reliably understand the world around us, and its achievements in biomedicine should not be undervalued.

Science is hard work with limited rewards and only occasional successes. Its interest and importance should speak for themselves, without hyperbole. Uncertainty is powerful and yet quite insufficiently acknowledged by either the working scientist or the journal editor who pretends prescience enough to more than guess at the ultimate value of todays endeavors. If, to return to Popper, "the striving for knowledge and the search for truth are still the strongest motives of scientific discovery [and if]...the advance of science depends upon the free competition of thought" (1), we in the biological and medical sciences must ask whether we have created a true market in scientific ideas that will serve this end.

**Acknowledgements.** Many colleagues have carefully read versions of this work, and the authors express their gratitude in particular to John Barrett (NHLBI), Rodrigo Calado (NHLBI), Cynthia Dunbar (NHLBI), Elihu Estey (University of Washington), Dean Follmann (NIAID), Peter Kostiuk (Logistics Management Institute), Jack Levin (University of California, San Francisco), Leonid Margolis (NICHD), Philip Mortimer (London), Alan Schechter (NIDDK), Philip Scheinberg (NHLBI), Colin Wu (NHLBI), Andrea Young (Columbia University), and Massimo Young (University of Chicago). ).

## References

[1] Popper K (2002) *The Logic of Scientific Discovery* (Routledge, New York).

[2] Arrow KJ (1962) *The Rate and Direction of Inventive Activity: Economic and Social Factors* (Princeton University Press, Princeton).

[3] Merton RK (1957) Priorities in scientific discovery: a chapter in the sociology of science. *American Sociological Review* 22:635–659.

[4] Lundvall B-A (1992) *National Systems of Innovation: Towards a Theory of Innovation and Interactive Learning* (Pinter Publishers, London).

[5] Nelson RR (1959) The simple economics of basic scientific research. *Journal of Political Economics* 67:297–306.

[6] Pavitt K (1991) What makes basic research economically useful? *Research Policy* 20:109-119.

[7] Polanyi M (1964) *Science, Faith and Society.* (University of Chicago Press, Chicago).

[8] Dasgupta P, David PA (1994) Toward a new economics of science. *Research Policy* 23:487–521.

[9] Stephan PE (1996) The economics of science. *Journal of Economic Literature* XXXIV:1199–1235.

[10] Stephan PE, Everhart SS (1998) The changing rewards to science: the case of biotechnology. *Small Business Economics* 10:141–151.

[11] Allison PD, Stewart JA (1974) Productivity differences among scientists: Evidence for accumulative advantage. *American Sociological Review* 39:596–606.

[12] Fox MF (1983) Publication productivity among scientists: a critical review. *Social Studies of Science* 13:285–305.

[13] Rosenberg N, Birdzell LE, Jr. (1990) Science, technology and the Western miracle. *Scientific American* 263:42–54.

[14] Johnson HG (1972) Some economic aspects of science. *Minerva* X:10–18.

[15] Callon M (1993) Is science a public good? *Science, Technology, and Human Values* 19:395–424.

[16] Rosenberg N (1974) Science, invention and economic growth. *Economic Journal* 84:90–108.

[17] Thaler RH (1988) Anomalies The Winner's Curse.*Journal of Economic Perspectives*. 12:191–202.

[18] Cox JC, Isaac RM (1984) In search of the winner's curse. *Economic Inquiry* 22:579–592.

[19] Dyer D, Kagel JH (1996) Bidding in common value auctions: how the commercial construction industry corrects for the winner's curse. *Management Science* 42:1463–1475.

[20] Harrison GW, List JA (2007) Naturally occurring markets and exogenous laboratory experiments: a case study of the winner's curse. *NBER Working Paper No 13072* 1–20.

[21] Capen EC, Clapp RV, Campbell WM (1971) Competitive bidding in high-risk situations. *Journal of Petroleum Technology* 23:641–653.

[22] Cassing J, Douglas RW (1980) Implications of the auction mechanism in baseball's free-agent draft. *Southern Economic Journal* 47:110-121.

[23] Dessauer JP (1981) *Book Publishing: What It Is, What It Does* (RR Bowker, New York).

[24] Hendricks K, Porter RH, Boudreau B (1987) Information, returns, and bidding behavior in OCS auctions: 1954–1969. *Journal of Industrial Economics* 35:517–542.

[25] Ioannidis JPA (2007) Contradicted and initially stronger effects in highly cited clinical research. *Journal of the American Medical Association* 2007 294:218–228.

[26] Krzyzanowska MK, Pintilie M, Tannock IF (2003) Factors associated with failure to publish large randomized trials presented at an oncology meeting. *Journal of the American Medical Association* 290:495–501.

[27] Stern JM (2006) Publication bias: evidence of delayed publication in a cohort study of clinical research projects. *British Medical Journal* 315:640–645.

[28] Goodman SN (2008) Systematic reviews are not biased by results from trials stopped early for benefit. *Journal of Clinical Epidemiology* 61:95–6.

[29] Bassler D, Ferreira-Gonzalez I, Briel M, Cook DJ, Devereaux PJ, et al. (2007) Systematic reviewers neglect bias that results from trials stopped early for benefit. *Journal of Clinical Epidemiology* 60:869–873.

[30] Ioannidis JP (2008). Why most discovered true associations are inflated. *Epidemiology* 19:640–648.

[31] Kavvoura FK, Liberpoulos G, Ioannidis JP (2008) Selection in reported epidemiological risks: an empirical assessment. *PLoS Medicine* 4:e79.

[32] Kyzas PA, Denaxa-Kyza D, Ioannidis JP (2007) Almost all articles on cancer prognostic markers report statistically significant results. *European Journal of Cancer* 43:2559–2579.

[33] Turner EH, Matthews AM, Linardatos E et al (2008) Selective publication of antidepressant trials and its influence on apparent efficacy. *New England Journal of Medicine* 358:252–260.

[34] Ioannidis JPA, Trikalinos TA (2005) Early extreme contradictory estimates may appear in published research: The Proteus phenomenon in molecular genetics research and randomized trials. *Journal of Clinical Epidemiology* 58:543–549.

[35] Ioannidis JPA (2005) Why most published research findings are false. *PLoS Medicine* 2:0696–0701.

[36] Simes RJ (1986) Publication bias: the case for an international registry of clinical trials. *Journal of Clinical Oncology* 4:1529–1541.

[37] Newcombe RG (1987) Towards a reduction in publication bias. *British Medical Journal* 29:656–659.

[38] Felson DT, Gantz L (2004) A surplus of positive trials: weighing biases and reconsidering equipoise. *Arthritis Research & Therapy* 6:117–119.

[39] Iyengar A, Greenhouse JB (1988) Selection models and the file drawer problem. *Statistical Science* 3:109–135.

[40] Scargle JD (2000) Publication bias: the "file-drawer" problem in scientific inference. *Journal of Scientific Exploration* 14:91–106.

[41] Greenwald AG (1975) Consequences of prejudice against the null hypothesis. *Psychological Bulletin* 82:1–20.

[42] Easterbrook P, et al (1991) Publication bias in clinical research. *Lancet* 337:867–872.

[43] Ioannidis JPA (2006) Evolution and translation of research findings: from bench to where? *PLoS Clinical Trials* 0001–0005.

[44] Contopoulos-Ioannidis DG, Ntzani E, Ioannidis JP (2003) Translation of highly promising basic science research into clinical applications. *American Journal of Medicine* 114:477–484.

[45] Schumpeter J (1928) The instability of capitalism. *Economic Journal* XXXVIII:361–386.

[46] The Cancer Genome Atlas. Accessed at: http://www.genome.gov/17516564. Last accessed July 9, 2008.

[47] Goodman SN, Altman DG, George SL (1998) Statistical reviewing policies of medical journals: caveat lector? *Journal of General Internal Medicine* 13: 753–756.

[48] Biomed Central: Most viewed articles in past year. Accessed at: http://www.biomedcentral.com/mostviewedbyyear/. Last accessed July 9, 2008.

[49] Frank RH, Cook PJ (1995) *The Winner-Take-All Society* (Free Press, New York).

[50] The PLoS Medicine Editors (2006) The impact factor game. *PLoS Medicine* 3:0001–0002.

[51] Smith R (2006) The power of the unrelenting impact factor – is it a force for good or harm? *International Journal of Epidemiology* 35:1129–1130.

[52] Andersen J, Belmont J, Cho CT (2006) Journal impact factor in the era of expanding literature. *Journal of Microbiology, Immunology, and Infection* 39:436–443.

[53] Ha TC, Tan SB, Soo KC (2006) The journal impact factor: too much of an impact? *Annals of the Academy of Medicine, Singapore* 35:911–916.

[54] Song F, Eastwood A, Bilbody S, Duley L (1999) The role of electronic journals in reducing publication bias. *Medical Informatics* 24:223–229.

[55] Rossner M, Van Epps H, Hill E (2007) Show me the data. *Journal of Experimental Medicine* 204:3052–3053.

[56] Chew M, Villanueva EV, van der Weyden MB (2007) Life and times of the impact factor: retrospective analysis of trends for seven medical journals (1994-2005) and their editors' views. *Journal of the Royal Society of Medicine* 100:142–150.

[57] Ronco C (2006) Scientific journals: who impacts the impact factor? *The International Journal of Artificial Organs* 29:645–648.

[58] Ioannidis JPA (2006) Concentration of the most-cited patpers in the scientific literature: analysis of journal ecosystems. *PLoS One* 1:1–7.

[59] Pan Z, et al (2005) Local literature bias in genetic epidemiology: an empirical evaluation of the Chinese literature. *PLoS Medicine* 2:1309–1317.

[60] Ireland NJ (1994) On limiting the market for status signals. *Journal of Public Economics* 53:91–110.

[61] Becker GS (1991) A note on restaurant pricing and other examples of social influences on price. *Political Economy* 99:1109–1116.

[62] Bikhchandani S, Hirshleifer D, Welch I (1998) Learning from the behavior of others: conformity, fads, and informational cascades. *Journal of Economic Perspectives* 12:151–170.

[63] Hirshleifer D, Teoh SH (2003) Herd behaviour and cascading in capital markets: a review and synthesis. *European Financial Management* 9:25–66.

[64] Rzhetsky A, et al (2007) Microparadigms: chains of collective reasoning in publications about molecular interactions. *Proceedings of the National Academy of Sciences of the USA* 103:4940–4945.

[65] Getting published in Nature: the editorial process. Accessed at: http://www.nature.com/nature/authors/get_published/index.html. Last accessed July 9, 2008

[66] About Science and AAAS. Accessed at: http://www.sciencemag.org/help/about/about.dtl. Last accessed July 9, 2008

[67] Bacon F (1989) *New Atlantis and the Great Instauration* (Harland Davidson, Wheeling IL).

[68] Calnan M, Smith GD, Sterne JAC (2006) The publication process itself

was the major cause of publication bias in genetic epidemiology. *Journal of Clinical Epidemiology* 59:1312–1318.

[69] Berlin JA (1992) Will publication bias vanish in the age of online journals? *Online Journal of Current Clinical Trials* 12.

[70] Littner Y, Mimouni FB, Dollberg S, Mandel D (2005) Negative results and impact factor *Archives of Pediatrics & Adolescent Medicine* 159:1036–1037.

[71] Olson CM, et al (2002) Publication bias in editorial decision making. *Journal of the American Medical Association* 287:2825–2828.

[72] Brown A, et al (2006) Association of industry sponsorship to published outcomes in gastrointestinal clinical research. *Clinical Gastroenterology and Hepatology* 4:1445–1451.

[73] Patsopoulos NA, Analatos AA, Ioannidis JPA (2006) Origin and funding of the most frequently cited papers in medicine: database analysis. *British Medical Journal* 332:1061–1064.

[74] Bubela TM, Caulfield TA (2004) Do the print media "hype" genetic research? A comparison of newspaper stories and peer-reviewed research papers. *Canadian Medical Association Journal* 170:1399–1407.

[75] Dickersin K, et al (1987) Publication bias and clinical trials. *Controlled Clinical Trials* 8:343–353.

[76] Callaham ML, et al (1998) Positive-outcome bias and other limitations in the outcome of research abstracts submitted to a scientific meeting. *Journal of the American Medical Association* 280:254–257.

[77] Kahneman D (2003) A perspective on judgment and choice: mapping bounded rationality. *American Psychologist* 58:697–720.

[78] Smith VL, Suchanek GL, Williams AW (1988) Bubbles, crashes and endogenous expectations in experimental spot asset markets. *Econometrica* 56:1119–1151.

[79] Kyzas PA, Loizou KT, Ioannidis JPA (2005) Selective reporting biases in cancer prognostic factor studies. *Journal of the National Cancer Institute* 97:1043–1055.

[80] Ioannidis JPA (2006) Journals should publish all "null" results and should sparingly publish "positive" results. *Cancer Epidemiology and Biomarkers & Prevention* 15:185.

**Table 1. Economic terms and analogies in scientific publication**

| Economic term | Meaning | Analogy in scientific publication |
|---|---|---|
| Winner's curse | The winner in an auction tends on average to have overpaid, especially when no participant is exactly certain of the item's real value. | Scientific studies try to find true relationships, but none is certain what these relationships are exactly. Published articles, especially in very competitive journals, have on average exaggerated results. |
| Oligopoly | A market where a few traders have the major share and each has significant power to influence the market. | Very few journals with limited publication slots (compared with geometrically increasing scientific data that seek publication) determine highly visible science. |
| Herding | "Follow-the-leader" behavior: the actions of the first or dominant player supersede the individual information and actions of all the players in a market. | Scientists may uncritically follow paths of investigation that are popularized in prestigious publications, neglecting novel ideas and truly independent investigative directions. |
| Artificial scarcity | Restrictions on the provision of a commodity above that expected from its production cost. | Print page limits are an obvious excuse for failure to accept articles, and further the small number of major "high impact" journals have limited slots; extremely low acceptance rates provide status signals to successful publications and their authors |
| Uncertainty | Situation where the real long-term value of a commodity is largely unpredictable. | For much–most?–scientific work, it is difficult or impossible to immediately predict future value, extensions, and practical applications. |
| Branding | Marking a product as valuable; of key importance when it is difficult to determine a product's value prior to consuming it. | Publishing in selective journals provides evidence of value of a research result and its authors, independent of the content of the manuscript. |

**Table 2. Potential competing or complementary options and solutions for scientific publication**

| | |
|---|---|
| 1 | Accept the current system as having evolved to be the optimal solution to complex and competing problems. |
| 2 | Promote rapid, digital publication of all articles that contain no flaws, irrespective of perceived "importance". |
| 3 | Adopt preferred publication of negative over positive results; require very demanding reproducibility criteria before publishing positive results. |
| 4 | Selection for publication in highly visible venues based on the quality of study methods, their rigorous implementation, and astute interpretation, irrespective of results. |
| 5 | Adopt formal post-publication downward adjustment of claims of papers published in prestigious journals. |
| 6 | Modify current practice to elevate and incorporate more expansive data to accompany print articles or to be accessible in attractive formats associated with high quality journals: combine the "magazine" and "archive" roles of journals. |
| 7 | Promote critical reviews, digests, and summaries of the large amounts of biomedical data now generated. |
| 8 | Offer disincentives to herding and incentives for truly independent, novel, or heuristic scientific work. |
| 9 | Recognize explicitly and respond to the branding role of journal publication in career development and funding decisions. |
| 10 | Modulate publication practices based on empirical research, which might address correlates of long-term successful outcomes (such as reproducibility, applicability, opening new avenues) of published papers. |
